# Supplementary material for: In Vitro Fermentation of Edible Mushrooms: Effects on Faecal Microbiota Characteristics of Autistic and Neurotypical Children
Source: Microorganisms. 2023 Feb 6;11(2):414. doi: 10.3390/microorganisms11020414 (PMC9959845; doi:10.3390/microorganisms11020414)
Supplement: Supplementary file 1 [file microorganisms-11-00414-s001.zip › microorganisms-2189672-supplementary.pdf]

microorganisms

Supplementary Material

In Vitro Fermentation of Edible Mushrooms: Effects on Faecal Microbiota Characteristics of Autistic and Neurotypical Children

Georgia Saxami [1], Evdokia K. Mitsou [1], Evangelia N. Kerezoudi [1,2], Ioanna Mavrouli [1], Marigoula Vlassopoulou [1], Georgios Koutrotsios [3], Konstantinos C. Mountzouris [4], Georgios I. Zervakis [3] and Adamantini Kyriacou [1,*]

[1] Department of Nutrition and Dietetics, Harokopio University, 17671 Athens, Greece; gsaxami@hua.gr (G.S.); emitsou@hua.gr (E.K.M.); dp4421804@hua.gr (E.N.K.); ds218031@hua.gr (I.M.); maryvlas@hua.gr (M.V.)
[2] School of Medical Sciences, Faculty of Medicine and Health, Örebro University, SE-701 82 Örebro, Sweden
[3] Laboratory of General and Agricultural Microbiology, Department of Crop Science, Agricultural University of Athens, 11855 Athens, Greece; georgioskoutrotsios@gmail.com (G.K.); zervakis@aua.gr (G.I.Z.)
[4] Department of Nutritional Physiology and Feeding, Agricultural University of Athens, 11855 Athens, Greece; kmountzouris@aua.gr
* Correspondence: mkyriacou@hua.gr; Tel.: +30-210-9549142

Table S1. Faecal microbial quantification per treatment between groups at the beginning of the fermentation (t=0 h).

	**Baseline (t=0h)**										**Overall *p***	
	NC		INU		scFOS		PO		PE		ASD	NT
	ASD	NT	ASD	NT	ASD	NT	ASD	NT	ASD	NT		
**Total bacteria**	9.94 (9.86-10.16)	9.96 (9.76-10.14)	9.95 (9.69-10.13)	9.87 (9.79-10.23)	9.86 (9.60-10.32)	9.95 (9.71-10.19)	9.80 (9.69-10.25)	9.76 (9.75-9.98)	9.79 (9.75-10.23)	9.99 (9.71-10.05)	0.948	0.771
***Bifidobacte-rium* spp.**	8.77 (7.34-9.23)	8.92 (8.58-9.46)	8.65 (7.39-9.22)	9.03 (8.58-9.51)	8.83 (7.28-9.26)	8.97 (8.61-9.51)	8.68 (7.25-9.25)	9.08 (8.40-9.43)	8.81 (7.39-9.26)	9.03 (8.57-9.56)	0.981	0.962
***Lactobacil-lus* group**	5.09 (4.96-5.69)	5.12 (4.75-6.36)	5.12 (4.95-5.61)	5.00 (4.81-6.47)	5.24 (4.86-5.67)	5.06 (4.85-6.47)	5.22 (4.93-5.61)	4.94 (4.77-6.46)	5.37 (4.98-5.64)	5.08 (4.91-6.48)	0.998	0.971
***C. perfringens* group**	6.55 (5.49-7.43)	6.92 (6.62-7.43)	6.52 (5.47-7.29)	7.10 (6.44-7.43)	6.39 (5.48-7.27)	6.92 (6.46-7.39)	6.84 (5.48-7.30)	7.12 (6.53-7.51)	6.87 (5.76-7.38)	6.98 (6.64-7.44)	0.988	0.965
***Bacteroides* spp.**	9.35 (8.90-9.61)	9.43 (9.01-9.76)	9.32 (8.86-9.66)	9.34 (9.05-9.75)	9.40 (8.66-9.72)	9.29 (9.06-9.75)	9.33 (8.96-9.65)	9.36 (8.95-9.74)	9.37 (8.96-9.68)	9.47 (9.06-9.64)	0.991	0.991
F. prausnitzii	8.56 (8.42-8.79)	8.63 (8.04-8.90)	8.61 (8.36-8.86)	8.69 (8.08-9.02)	8.51 (8.31-8.81)	8.54 (8.02-8.94)	8.56 (8.47-8.77)	8.56 (8.01-8.94)	8.53 (8.46-8.81)	8.77 (8.03-8.93)	0.974	0.991
***Prevotella* spp.**	6.49 (6.42-8.16)[†]	9.34 (8.57-9.55)	6.43 (6.36-8.10)	9.33 (8.55-9.75)	6.58 (6.14-8.20)	9.27 (8.56-9.69)	6.52 (6.41-8.10)	9.18 (8.35-9.61)	6.64 (6.47-8.16)	9.24 (8.53-9.61)	0.898	0.975
A. mucini-phila	6.75 (5.10-8.94)	4.55 (4.22-8.33)	6.64 (5.15-9.01)	4.51 (4.29-8.33)	6.79 (4.87-9.05)	4.44 (4.28-8.26)	6.76 (5.00-9.01)	4.30 (4.11-8.32)	6.74 (5.21-9.03)	4.36 (4.20-8.27)	0.999	0.963

Values are expressed as median and Q1–Q3 quartiles for nonparametric data . †:significantly different compared to neurotypical children in the case of each treatment (NC, INU, scFOS, PO and PE) after 24h fermentation (Mann Whitney test). Quantitative PCR (qPCR); log_{10} copies of 16S rRNA gene mL^{-1} of sample. ASD: Children with autism spectrum disorders, NT: Neurotypical children

Table S2: % Faecal microbial median changes in ASD children's faeces for each investigated substrate

	**NC**	**INU**	**scFOS**	**PO**	**PE**	**Overall *p***
			**% Δ changes**			
**Total bacteria**	1.02 (0.01-2.59)	3.94 (2.27-6.72)	5.09 (1.50-7.98)	7.25 (0.69-8.20)	8.38 (2.52-9.93)	0.196
***Bifidobacterium* spp.**	0.82 (0.21-2.52)	13.94 (6.16-16.44)*	14.81 (7.56-16.55)*	7.50 (5.02-13.50)	8.02 (6.71-11.52)	0.009
***Lactobacillus* group**	-0.29 (-4.61 - 1.03)	-0.41 (-1.79 - 7.93)	-1,41 (-2.49 - 7.41)	1.99 (-1.87 - 5.41)	0.71 (-1.53 - 11.55)	0.796
C. perfigens group	-0.36 (-3.85 - 2.03)	3.93 (-0.46 - 7.26)	4.97 (-2.69 - 14.32)	4.07 (0.82 - 5.47)	6.68 (-1.54 - 18.38)	0.619
***Bacteroides* spp.**	-0.42 (-3.40 - 4.91)	4.45 (2.36 - 7.58)	3.52 (1.75 - 9.32)	4.56 (1.27 - 7.46)	7.14 (3.18 - 9.84)	0.201
F. prausnitzii	-1.35 (-4.11 - 2.95)	6.20 (1.70-6.88)	4.18 (1.15-9.54)	5.43 (1.40-9.52)	8.18 (2.24 - 12.65)	0.169
***Prevotella* spp.**	2.41 (-7.79-7.07)	7.88 (2.91-10.78)	10.88 (2.78-15.35)	7.21 (2.52-10.28)	10.23 (3.18-13.32)	0.460
A. muciniphila	9.46 (0.52-30.38)	5.65 (-0.39-7.79)	-0.60 (-0.79-16.14)	1.56 (-0.45-17.05)	6.83 (-0.32-13.57)	0.721
**24h-Prebiotic Indexes**	0.09 (-1.16 - 0.66)	4.59 (0.18-8.85)	3.53 (-0.36 - 10.38)	0.60 (-0.58 - 1.87)	-0.21 (-2.61 - 3.25)	0.237

Values are expressed as median and Q1-Q3 quartiles for non parametric data where % Δ changes are defined as '(Median log_{10} copies/ml sample of each substrate at t=24 minus median log_{10} copies/ml sample of each substrate at t=0)/ median log_{10} copies/ml sample of each substrate at t=0) * 100'.

Table S3: % Faecal microbial median changes in neurotypical children's faeces for each investigated substrate

	**NC**	**INU**	**scFOS**	**PO**	**PE**	**Overall *p***
			**% Δ changes**			
**Total bacteria**	-2.21 (-2.62 - 1.76)	5.12 (0.54 - 5.63)	2.59 (1.56-7.02)	5.11 (3.63-7.37)	5.78 (2.44-8.31)	
***Bifidobacterium* spp.**	0.33 (-0.19 - 0.56)	10.92 (8.23-16.22)*	12.91 (7.96-19.33)*	8.14 (5.26-12.59)	7.33 (3.94-10.55)	0.034

Lactobacillus group	-0.50 (-0.80 - 0.23)	0.38 (-4.19 - 6.01)	1.17 (-6.80 - 3.42)	-0.08 (-3.50 - 5.73)	1.51 (-4.23- 5.62)	0.006
***C. perfigens* group**	-2.24 (-4.20 - 1.95)	1.50 (-1.05 - 13.22)	3.05 (-0.73 - 10.49)	-0.05 (-5.62 - 10.50)	-0.51 (-3.37 - 18.25)	0.972
***Bacteroides* spp.**	-2.84 (-5.81 - 2.96)	5.52 (2.86-8.19)	4.96 (1.80-7.15)	5.86 (2.97-10.41)	4.94 (2.23-8.24)	0.466
F. prausnitzii	-5.79 (-13.33 - -3.48)	-0.19 (-1.56 - 0.27)	-0.23 (-0.99 - 1.30)	3.57 (1.49-6.32)*	1.18 (-0.03 - 9.61)*	0.129
***Prevotella* spp.**	-14.69 (-18.84 - -7.92)	5.72 (2.18-8.61)*	4.80 (0.89-5.43)	2.61 (-3.89 - 9.20)	0.24 (-4.17 - 5.25)	0.001
A. muciniphila	5.56 (-1.12 - 10.28)	-0.39 (-6.24 - 4.62)	0.56 (-4.28 - 4.60)	8.77 (-0.64 - 11.53)	3.26 (0.65 - 7.73)	0.031
**24h-Prebiotic Indexes**	1.30 (-1.09 - 1.89)	2.13 (1.82 - 5.96)	4.12 (2.13 - 11.19)	0.29 (-2.09 - 1.65)	-0.18 (-5.03 - 2.43)	0.322

Values are expressed as median and Q1-Q3 quartiles for non parametric data where % Δ changes are defined as '(Median log_{10} copies/ml sample of each substrate at t=24 minus median log_{10} copies/ml sample of each substrate at t=0)/ median log_{10} copies/ml sample of each substrate at t=0) * 100'.

Table S4: Prebiotic Indexes (PIs) per subject for each one of the treatments included in this study

Prebiotic Indexes										
	**ASD subjects**					**NT subjects**				
Treatment	no1	no2	no3	no4	no5	no1	no2	no3	no4	no5
NC	-1.42	-0.36	ND	0.70	0.54	2.04	1.30	-2.75	0.57	1.74
INU	4.88	-1.19	ND	4.30	10.17	5.32	1.69	6.61	2.13	1.95
scFOS	4.41	-1.36	ND	2.64	12.37	8.12	2.10	14.26	4.12	2.15
PO	1.32	-0.74	ND	-0.12	2.05	0.70	-1.00	-3.18	2.59	0.29
PE	-3.02	-1.36	ND	4.01	0.95	-0.67	-0.18	-8.40	2.67	2.19

NC: Negative control; INU: inulin; scFOS: short-chain fructooligosaccharides; PO: *Pleurotus ostreatus* IK 1123; PE: *Pleurotus eryngii* LGAM 216.ASD subjects: Children with autism spectrum disorders, NT subjects: Neurotypical children

Table S5. Total short-chain fatty acids (TSCFAs) and SCFAs concentrations (μmol mL^{-1} of sample), at the beginning of the fermentation for the ASD and neurotypical group

**Concentration μmol/ml**												
	**NC**		**INU**		**scFOS**		**PO**		**PE**		**Overall *p* value**	
	ASD	NT	ASD	NT	ASD	NT	ASD	NT	ASD	NT	ASD	24h
TSCFAs	5.66 (4.97-7.02)	3.80 (2.93-3.94)	3.16 (2.49-4.31)	2.00 (1.35-3.31)	2.38 (1.97-4.02)	1.80 (1.40-2.56)	2.74 (1.91-4.06)	1.92 (1.48-2.63)	2.50 (1.81-3.82)*	1.91 (1.34-2.77)	0.024	0.101
AA	2.95 (2.22-3.74)	1.73 (1.24-2.08)	1.55 (1.31-2.63)	0.98 (0.62-1.74)	1.25 (1.10-2.40)	0.83 (0.67-1.15)	1.50 (1.16-2.67)	1.08 (0.77-1.27)	1.40 (1.09-2.48)	1.06 (0.69-1.35)	0.159	0.114
PPA	1.03 (0.63-1.13)	0.79 (0.52-0.87)	0.45 (0.39-0.50)	0.43 (0.24-0.65)	0.36 (0.31-0.39)*	0.37 (0.33-0.54)	0.38 (0.27-0.41)*	0.41 (0.22-0.53)	0.34 (0.27-0.39)*	0.40 (0.20-0.62)	0.003	0.228
BA	1.93 (0.73-2.65)	0.98 (0.51-1.03)	0.71 (0.54-0.99)	0.40 (0.28-0.50)	0.38 (0.34-0.71)	0.38 (0.18-0.45)	0.32 (0.25-0.66)*	0.36 (0.16-0.45)	0.29 (0.24-0.64)*	0.41 (0.14-0.43)*	0.009	0.028
BSCFAs	0.18 (0.13-0.21)	0.16 (0.10-0.20)	0.15 (0.14-0.17)	0.14 (0.10-0.23)	0.13 (0.13-0.26)	0.13 (0.09-0.21)	0.13 (0.13-0.21)	0.15 (0.11-0.19)	0.13 (0.12-0.19)	0.15 (0.10-0.20)	0.750	0.975
Other	0.11 (0.09-0.20)	0.15 (0.10-0.21)	0.09 (0.08-0.16)	0.09 (0.08-0.21)	0.10 (0.09-0.36)	0.12 (0.08-0.25)	0.09 (0.09-0.27)	0.11 (0.08-0.24)	0.09 (0.09-0.24)	0.11 (0.08-0.22)	0.938	0.957

Values are expressed as median and Q1-Q3 quartiles for non parametric data; *: significantly different compared to NC (Kruskall Wallis test with pairwise comparisons); AA: acetate, PPA: Propionate, BA: butyrate, BSCFAs: Branched short-chain fatty acids. ASD: Children with autism spectrum disorders, NT: Neurotypical children

Table S6: Differences (Δ) in SCFAs concentrations (μmol mL^{-1}) after 8h (ΔC_{t8-0}) and 24h of fermentation (ΔC_{t24-0}) for the ASD children group

			SCFAs production rates			
SCFAs production rates	NC	INU	scFOS	PO	PE	Overall *p*
			ΔC ΔC_{t8-0}			
TVFAs	6.93 (4.79-12.47)	25.92 (19.69-37.37)	29.15 (24.61-43.26)	34.00 (28.35-41.06)	45.26 (33.89-48.54)*	0.005
Acetate	5.88 (3.39-8.40)	16.38 (14.36-28.47)	21.88 (17.17-36.13)	24.72 (18.58-25.18)	25.41 (22.32-34.58)*	0.012
Propionate	1.53 (0.61-1.81)	3.83 (1.84-4.25)	3.15 (1.68-4.90)	6.60 (3.30-7.03)	7.54 (3.00-10.89)*	0.019
Butyrate	-0.15 (-1.03 - 1.83)	3.79 (2.37-6.15)	4.69 (1.81-5.79)	7.11 (3.01-9.62)*	6.34 (2.48-11.16)*	0.021
BSCFAs	0.39 (0.22-1.07)	0.09 (0.04-0.18)	0.04 (0.02-0.07)*	0.10 (0.08-0.15)	0.17 (0.11-0.45)	0.003
other	0.24 (0.10-0.38)	0.10 (0.06-0.20)	0.07 (-0.18-0.15)	0.05 (-0.05-0.23)	0.04 (-0.03-0.35)	0.433
			ΔC_{t24-0}			
TVFAs	18.48 (16.40-22.54)	45.49 (36.22-62.20)	60.79 (44.06-76.04)	57.98 (49.59-65.46)	75.93 (58.91-81.00)*	0.004
Acetate	12.43 (9.01-13.96)	32.57 (22.04-49.77)	51.04 (32.85-68.19)*	28.84 (26.55-35.28)	37.13 (29.39-51.02)*	0.009
Propionate	2.67 (2.10-3.49)	5.44 (2.76-5.72)	3.51 (2.03-5.90)	9.36 (8.96-11.75)*	12.38 (9.41-17.57)*	0.001
Butyrate	1.24 (0.24-4.18)	8.64 (4.89-10.90)	5.79 (2.16-9.04)	17.16 (9.90-19.93)*	19.70 (7.10-25.60)*	0.006
BSCFAs	2.07 (2.04-2.17)	0.34 (0.27-0.77)§	0.04 (-0.02-0.13)*	0.46 (0.17-2.12)	0.78 (0.16-2.12)	0.002
other	0.69 (0.64-0.81)	0.17 (0.10-0.36)	0.09 (-0.19-0.20)	0.15 (0.05-1.34)	0.19 (0.11-1.05)	0.052

Values are expressed as median and Q1-Q3 quartiles for non parametric data where ΔC_{t8-0} is defined as '[(Concentration t=8h minus Concentration t=0h)/ Concentration t=0h]' and ΔC_{t24-0} is as '[(Concentration t=24h minus Concentration t=0h)/ Concentration t=0h]'. *: significantly different compared to NC after 24h of fermentation for ASD children (Kruskall Wallis test with paiwise comparisons); §: significantly different compared to neurotypical children in the case of each treatment (NC, INU, scFOS, PO and PE) after fermentation (Mann Whitney test). NC: Negative control; INU: inulin; scFOS: short-chain fructooligosaccharides; PO: *Pleurotus ostreatus* IK 1123; PE: *Pleurotus eryngii* LGAM 216.

Table S7: Differences (Δ) in SCFAs concentrations (μmol mL^{-1}) after 8h (ΔC_{t8-0}) and 24h of fermentation (ΔC_{t24-0}) for the neurotypical children group

			SCFAs production rates			
	NC	INU	scFOS	PO	PE	Overall *p*
			ΔC_{t8-0}			
TVFAs	8.05 (4.31-10.39)	33.21 (17.90-43.06)	32.57 (19.54-46.39)	31.34 (20.55-44.28)	37.44 (20.90-59.54)*	0.017
Acetate	5.13 (3.31-6.61)	20.41 (13.51-28.03)	24.24 (15.03-34.15)*	22.09 (15.64-26.00)	24.40 (16.04-31.14)*	0.018
Propionate	1.29 (0.61-2.32)	3.81 (1.41-14.44)	3.50 (1.43-11.17)	4.28 (2.06-11.92)	4.54 (2.11-22.17)	0.209
Butyrate	0.08 (-0.15 - 1.26)	3.27 (1.77-4.19)	2.65 (1.02-3.96)	4.34 (2.18-6.59)	4.36 (2.28-8.15)	0.053
BSCFAs	0.30 (0.18-0.52)	0.01 (-0.07 - 0.15)	0.00 (-0.08 - 0.13)	0.08 (0.02-0.19)	0.09 (0.02-0.17)	0.056
other	0.26 (0.14-0.39)	0.12 (0.08-0.25)	0.07 (-0.03 - 0.19)	0.17 (0.12-0.30)	0.15 (0.11-0.21)	0.243
			ΔC_{t24-0}			
TVFAs	18.62 (17.31-20.39)	54.75 (48.20-79.86)	71.34 (69.61-77.59)	68.21 (55.88-72.79)	79.62 (74.63-81.83)*	0.003
Acetate	11.37 (9.72-12.00)	41.80 (26.79-62.88)	66.96 (51.58-68.26)*	37.55 (32.22-38.89)	43.70 (34.93-49.08)	0.002
Propionate	2.08 (1.83-4.25)	6.81 (5.28-19.09)	4.88 (3.45-13.72)	10.94 (8.46-19.45)*	16.28 (9.65-27.00)*	0.007
Butyrate	1.64 (1.16-2.88)	4.41 (3.55-10.54)	3.14 (1.33-6.69)	13.79 (10.10-17.20)*	16.07 (14.18-20.76)*,†	0.001
BSCFAs	2.17 (2.01-2.34)	0.06 (0.01-0.24)*	0.00 (-0.06 - 0.15)*	0.29 (0.13-2.17)	0.19 (0.06-0.77)	0.007
other	0.97 (0.62-1.09)	0.23 (0.09-0.33)	0.10 (-0.01 - 0.21)*	0.71 (0.36-2.16)†	0.39 (0.38-1.17)	0.001

Values are expressed as median and Q1-Q3 quartiles for non parametric data where ΔC_{t8-0} is defined as '[(Concentration t=8h minus Concentration t=0h)/ Concentration t=0h]' and ΔC_{t24-0} is as '[(Concentration t=24h minus Concentration t=0h)/ Concentration t=0h]' *: significantly different compared to NC after 24h of fermentation for neurotypical children (Kruskall Wallis test with paiwise comparisons); †: significantly different compared to scFOS after fermentation for neurotypical children (Kruskall Wallis test with paiwise comparisons). NC: Negative control; INU: inulin; scFOS: short-chain fructooligosaccharides; PO: *Pleurotus ostreatus* IK 1123; PE: *Pleurotus eryngii* LGAM 216

Table S8: % Differences (Δ) in SCFAs concentrations (μmol mL^{-1}) after 8h (ΔC_{t8-0}) and 24h of fermentation (ΔC_{t24-0}) for the ASD children group

	% SCFAs production rates					
	**NC**	**INU**	**scFOS**	**PO**	**PE**	**Overall p**
			**%ΔC_{t8-0}**			
TVFAs	154.94 (75.20-212.14)	719.84 (637.42-1170.68)§	1240.45 (835.68-1718.86)*	1239.59 (971.88-1654.21)*	1854.77 (1180.65-2130.36)*	0.003
Acetate	233.05 (110.82-284.98)	1199.56 (785.41-1555.32)	1751.66 (1032.01-2558.34)*	1462.31 (904.35-1956.66)	2172.58 (1084.45-2621.69)*	0.009
Propionate	148.80 (54.16-289.52)	700.10 (433.96-1066.90)	882.32 (450.85-1437.15)	1719.78 (929.90-2070.08)*	2213.76 (974.41-3297.05)*	0.004
Butyrate	-7.86 (-37.37-248.83)	447.61 (258.33-1191.95)	595.09 (476.34-1318.47)	1227.34 (996.60-2214.84)*	1264.47 (893.55-2639.61)*	0.005
BSCFAs	260.81 (158.91-566.88)	67.34 (28.22-106.16)	14.52 (11.27-53.94)*	59.31 (53.88-95.60)	135.15 (81.07-224.59)	0.003
other	184.11 (54.27-407.18)	99.92 (40.53-258.12)	78.99 (-24.94-160.94)	58.52 (-1.71-272.29)	48.40 (-3.07-398.41)	0.607
			**%ΔC_{t24-0}**			
TVFAs	348.20 (250.87-418.93)§	1479.75 (1003.14-2092.94)	2774.15 (1190.03-3468.76)*	2050.76 (1603.23-2856.48)	2826.23 (2215.30-3537.45)*	0.005
Acetate	359.06 (293.66-562.47)	2200.34 (1035.99-3038.65)	4465.17 (1372.10-5513.82)*	1971.41 (1386.72-2410.35)§	2479.73 (1745.75-3712.17)*	0.009
Propionate	236.55 (196.12-552.60)	1082.30 (647.15-1376.36)§	982.35 (540.20-1734.71)	3068.36 (2304.05-3608.67)*	4920.61 (2596.90-5400.41)*	0.001
Butyrate	64.11 (10.45-577.10)	1072.13 (567.85-1673.45)	1072.13 (567.85-1673.45)	3306.01 (2686.33-5735.70)*	4638.12 (2285.75-6721.08)*	0.001
BSCFAs	1162.05 (1008.51-1673.14)	239.21 (167.62-500.94)§	30.66 (0.69-96.04)*	228.23 (117.36-1585.75)	594.67 (122.37-848.99)	0.004
other	558.21 (353.60-932.50)	189.42 (67.98-465.39)	96.91 (-12.30-211.70)	150.09 (70.17-1549.47)	173.18 (62.80-1168.50)	0.115
TVFAs	348.20 (250.87-418.93)§	1479.75 (1003.14-2092.94)	2774.15 (1190.03-3468.76)*	2050.76 (1603.23-2856.48)	2826.23 (2215.30-3537.45)*	0.005

Values are expressed as median and Q1-Q3 quartiles for non parametric data where %ΔC_{t8-0} is defined as '[(Concentration t=8h minus Concentration t=0h)/ Concentration t=0h]*100' and ΔC_{t24-0} is as '[(Concentration t=24h minus Concentration t=0h)/ Concentration t=0h]*100' *: significantly different compared to NC after 24h of fermentation for ASD children (Kruskall Wallis test with pairwise comparisons); §: significantly different compared to neurotypical children in the case of each treatment (NC, INU, scFOS, PO, PE) after fermentation (Mann Whitney test). NC: Negative control; INU: inulin; scFOS: short-chain fructooligosaccharides; PO: *Pleurotus ostreatus* IK 1123; PE: *Pleurotus eryngii* LGAM 216

Table S9: % Differences (Δ) in SCFAs concentrations (μmol mL^{-1}) after 8h (ΔC_{t8-0}) and 24h of fermentation (ΔC_{t24-0}) for the neurotypical children group

	% SCFAs production rates					
	**NC**	**INU**	**scFOS**	**PO**	**PE**	**Overall p**
			**%ΔC_{t8-0}**			
TVFAs	229.18 (150.71-264.30)	1340.10 (1109.89-1878.10)	1484.08 (1274.83-2375.85)	1784.06 (1180.82-1940.49)	1845.60 (1334.44-2812.48)*	0.012
Acetate	313.40 (245.09-333.89)	2305.27 (1436.41-2801.59)	2702.55 (1757.34-4134.83)*	2124.36 (1594.21-2598.15)	2294.29 (1707.80-3394.86)	0.016
Propionate	202.63 (118.04-290.37)	876.93 (584.77-2458.35)	819.58 (454.42-2234.13)	1123.70 (847.83-2365.36)	1204.91 (974.48-4280.75)*	0.021
Butyrate	9.72 (-13.68-218.05)	809.39 (476.73-996.23)	615.40 (530.33-929.34)	1005.29 (772.94-2188.18)*	1034.86 (724.42-2842.15)*	0.007
BSCFAs	272.09 (124.14-278.70)	15.88 (-31.57 - 89.55)	0.36 (-38.17 - 100.56)	56.84 (19.05-108.49)	96.17 (9.92-115.98)	0.038
other	197.30 (107.35-230.88)	131.62 (70.99-225.16)	76.37 (9.01-193.77)	177.55 (70.26-256.54)	122.79 (71.36-214.96)	0.640
			**%ΔC_{t24-0}**			

TVFAs	534.15 (464.00-653.35)	3485.46 (2183.02-4208.34)	3823.25 (2913.57-5479.85)*	3015.19 (2678.44-4432.03)	4208.17 (2859.96-6387.16)*	0.015
Acetate	621.43 (483.90-970.34)	5801.78 (2823.78-6794.92)	6938.44 (5099.42-10750.45)*	3233.50 (3005.49-4746,19)	3904.41 (2653.21-7885.62)	0.005
Propionate	500.33 (241.48-534.00)	2184.12 (1680.62-3658.01)	1633.43 (862.46-2660.13)	4256.33 (2289.63-5027.46)*	6442.25 (2145.62-7913.05)*	0.004
Butyrate	263.97 (118.21-480.11)	1058.13 (783.09-3284.31)	874.59 (653.47-1554.51)	4510.77 (3275.21-7053.13)*	5569.62 (4012.14-10355.36)*	0.001
BSCFAs	1433.69 (1196.47-1987.03)	56.52 (14.32-152.75)*	3.38 (-28.73 - 120.87)*	298.62 (78.34-1162.11)	204.98 (39.34-486.29)	0.005
other	532.56 (445.99-843.73)	184.65 (84.16-323.25)	83.55 (25.88-210.07)*	620.55 (276.50-1739.59)	423.48 (251.95-833.89)	0.010

Values are expressed as median and Q1-Q3 quartiles for non parametric data where % ΔC_{t8-0} is defined '[(Concentration t=8h minus Concentration t=0h)/ Concentration t=0h] *100' and ΔC_{t24-0} is as '[(Concentration t=24h minus Concentration t=0h)/ Concentration t=0h] *100' *: significantly different compared to NC after 24h of fermentation for ASD children (Kruskall Wallis test with pairwise comparisons). NC: Negative control; INU: inulin; scFOS: short-chain fructooligosaccharides; PO: *Pleurotus ostreatus* IK 1123; PE: *Pleurotus eryngii* LGAM 216
